# Acute Hypercapnic Respiratory Failure as the Initial Presentation of Motor Neuron Disease: A Respiratory Diagnostic Challenge

**DOI:** 10.7759/cureus.112832

**Published:** 2026-07-17

**Authors:** Shayekh Ferdoush, Mohamed Seklani, Prasad Kumar, Sureni Rosamalika Jayarathne Abekoon Mudiyanselage, Anvia Dsouza

**Affiliations:** 1 Respiratory Medicine, University Hospitals Bristol and Weston NHS Foundation Trust, Weston-super-Mare, GBR; 2 Pulmonology, University Hospitals Bristol and Weston NHS Foundation Trust, Bristol, GBR; 3 Medicine, University Hospitals Bristol and Weston NHS Foundation Trust, Weston-super-Mare, GBR

**Keywords:** amyotrophic lateral sclerosis (als), british thoracic society (bts), motor neuron disease, noninvasive ventilation (niv), type 2 respiratory failure (t2rf)

## Abstract

Amyotrophic lateral sclerosis (ALS) is a progressive neurodegenerative disorder characterized by degeneration of the upper and lower motor neurons, with respiratory muscle involvement typically occurring in the later stages of the disease. Presentation with acute hypercapnic respiratory failure as the initial clinical manifestation is uncommon and frequently leads to diagnostic delay or misattribution to primary cardiopulmonary pathology. We present the case of a 60-year-old man with no prior respiratory history who presented with acute dyspnea and was found to have severe hypercapnic respiratory failure requiring urgent noninvasive ventilation. Clinical examination and initial investigations revealed no clear intrinsic pulmonary cause. A detailed history subsequently identified a four-month progression of neurological symptoms, including dysarthria, sialorrhea, asymmetric upper limb weakness, and significant unintentional weight loss. Neurological examination demonstrated tongue fasciculations, widespread muscle wasting, and a combination of upper and lower motor neuron signs. Following acute stabilization and discharge with domiciliary noninvasive ventilation, a specialist neurological assessment confirmed a diagnosis of ALS. This case highlights the clinical importance of considering underlying neuromuscular causes in patients presenting with unexplained hypercapnic respiratory failure, particularly when clinical findings are discordant with the severity of the gas exchange abnormalities. Early recognition of ALS in this context facilitates the timely initiation of ventilatory support and multidisciplinary care, which are essential for optimizing survival and quality of life.

## Introduction

Type 2 respiratory failure results from alveolar hypoventilation and is most commonly associated with conditions such as chronic obstructive pulmonary disease (COPD) and obesity hypoventilation syndrome (OHS) [1,2]. However, neuromuscular disorders represent an important and frequently underrecognized cause of ventilatory failure.

Motor neuron disease (MND) is characterized by progressive degeneration of the upper and lower motor neurons. In amyotrophic lateral sclerosis (ALS), respiratory muscle weakness initially leads to nocturnal hypoventilation and subsequently to daytime hypercapnia, which is a major determinant of prognosis [3,4]. Presentation with acute ventilatory failure as the first clinical manifestation is rare and may lead to misdiagnosis or delayed recognition [5].

## Case presentation

A 60-year-old previously independent man presented with acute-onset dyspnea. He denied cough, sputum production, fever, wheeze, or chest pain. There was no history of chronic respiratory disease. Arterial blood gas analysis on room air demonstrated a pH of 7.21, PaCO₂ of 14.0 kPa, PaO₂ of 7.6 kPa, bicarbonate of 37.1 mmol/L, and oxygen saturation of 85.3%. These findings indicated severe hypercapnic respiratory acidemia. The markedly elevated bicarbonate concentration suggested chronic renal compensation for sustained hypoventilation, indicating that chronic hypercapnia was likely present before the acute decompensation, although previous arterial blood gas measurements were unavailable to confirm this. The calculated alveolar-arterial oxygen gradient was approximately 1.3 kPa while breathing room air, supporting alveolar hypoventilation rather than intrinsic parenchymal lung disease. The patient required urgent initiation of noninvasive ventilation, with subsequent clinical and biochemical improvement.

He was an ex-smoker with a 23-pack-year history, having stopped smoking 12 years previously, and denied vaping. His medical history included hiatus hernia, heart failure with preserved ejection fraction, and aortic stenosis. On examination, the chest was clear to auscultation, with no wheeze or crackles, but he was dyspneic while speaking. There was no evidence of fluid overload, and the jugular venous pressure was not elevated. Despite minimal respiratory findings, the patient exhibited significant oxygen desaturation, with oxygen saturation falling to 70-80%. This marked discordance between the clinical findings and the severity of the gas exchange abnormality raised suspicion of an extrapulmonary cause of ventilatory failure. Further history revealed progressive neurological symptoms over the preceding four months. These included dysarthria, sialorrhea, more prominent on the left, progressive left upper limb weakness, fluctuating concentration, and significant unintentional weight loss of approximately two stone over 12 months, associated with early satiety.

Neurological examination demonstrated preserved higher cortical function. Speech was dysarthric but fully intelligible. There was no ptosis, and extraocular movements were normal. Tongue fasciculations were present with relatively preserved mobility, and the jaw jerk was brisk. There was generalized muscle wasting involving all four limbs, including the intrinsic hand muscles and pectoral muscles, with visible hand muscle guttering. Widespread fasciculations were observed involving the tongue, shoulder girdle, back, and lower limbs. Multifocal upper limb weakness was present, with mildly increased tone and brisk reflexes despite muscle wasting. Plantar responses were flexor. Cerebellar function and gait were preserved.

Routine laboratory investigations, autoimmune screening, infectious serology, echocardiography, and cross-sectional imaging did not identify an alternative metabolic, autoimmune, infectious, structural, or cardiopulmonary explanation for the patient’s presentation (Table 1).

**Table 1:** Summary of laboratory, autoimmune, infectious, cardiac, and imaging investigations. AChR, acetylcholine receptor; ANA, antinuclear antibody; ANCA, antineutrophil cytoplasmic antibody; CCP, cyclic citrullinated peptide; ENA, extractable nuclear antigen antibody; MuSK, muscle-specific kinase; RCC, right coronary cusp; NCC, noncoronary cusp; TSH, thyroid-stimulating hormone

Investigation	Findings	Reference range/normal values
CRP	3 mg/L	<5 mg/L
Hemoglobin	143 g/L	130-170 g/L
HIV-1 and HIV-2	Negative	-
Anti-voltage-gated calcium channel antibody	Negative	-
Anti-AChR antibody	Negative	-
AChR antibody by indirect immunofluorescence	Negative	-
MuSK antibody by indirect immunofluorescence	Negative	-
Total syphilis antibody	Negative	-
Vitamin B12	206 ng/L	180-900 ng/L
TSH	2.6 mIU/L	0.38-5.33 mIU/L
Autoimmune profile, including ANA, ANCA, ENA profile, rheumatoid factor, and anti-CCP antibody	Negative	-
Transthoracic echocardiogram	Mild-to-moderate calcific aortic stenosis. Possible bicuspid aortic valve with RCC/NCC fusion, although this could not be confirmed. Concentric remodeling with increased mid-septal wall thickness (~14 mm). Borderline low left ventricular systolic function without dilatation. Estimated left ventricular ejection fraction by the biplane Simpson method: 54%. Impaired diastolic function with normal filling pressures. Normal right ventricular size and function. Normal-sized atria.	
Contrast-enhanced CT of the neck, thorax, abdomen, and pelvis	No structural abnormality explaining the patient’s presentation was identified.	

The patient was stabilized with noninvasive ventilation and discharged with domiciliary ventilatory support under respiratory follow-up. Subsequent neurological review confirmed a diagnosis of ALS based on clinical evidence of combined upper and lower motor neuron involvement, consistent with contemporary diagnostic criteria.

## Discussion

This case highlights an atypical but clinically significant presentation of MND encountered in respiratory practice. The key diagnostic challenge lies in recognizing neuromuscular ventilatory failure in the absence of intrinsic lung disease.

From a physiological perspective, hypercapnic respiratory failure in ALS results from progressive diaphragmatic and respiratory muscle weakness, leading to reduced tidal volumes and impaired alveolar ventilation [4]. Patients typically develop nocturnal hypoventilation before progressing to daytime hypercapnia. The relatively preserved alveolar-arterial oxygen gradient distinguishes hypoventilation from parenchymal lung disease.

The differential diagnosis of hypercapnic respiratory failure includes COPD, OHS, myasthenia gravis, and Guillain-Barré syndrome. In this case, the absence of airflow limitation, parenchymal abnormalities, or cardiac decompensation made common cardiopulmonary causes unlikely. The presence of progressive bulbar dysfunction and combined upper and lower motor neuron signs was pivotal in establishing the diagnosis.

The diagnosis of ALS is primarily clinical and may be assessed using the contemporary Gold Coast diagnostic criteria, which require progressive motor impairment together with evidence of upper and lower motor neuron dysfunction after exclusion of alternative diagnoses [6]. Diagnostic delay is common, particularly in atypical presentations, and may adversely affect timely access to supportive therapies.

Noninvasive ventilation is a cornerstone of management in ALS-related respiratory failure. Randomized controlled trials have demonstrated that noninvasive ventilation improves both survival and quality of life, particularly when initiated before advanced respiratory compromise [7]. Current recommendations from the National Institute for Health and Care Excellence and the British Thoracic Society advocate early respiratory assessment and timely initiation of ventilatory support in patients with neuromuscular disease [8-10].

This case underscores the importance of maintaining a high index of suspicion for neuromuscular causes in patients with unexplained hypercapnic respiratory failure. Careful history-taking and neurological examination remain critical components of the assessment.

Limitations

This report has several limitations. Formal respiratory muscle assessments, including spirometry, maximal inspiratory and expiratory pressures, and sniff nasal inspiratory pressure measurements, were not available during the acute admission because the patient was unable to complete reliable testing. Similarly, detailed longitudinal respiratory physiological data and complete noninvasive ventilation parameters were not available for reporting. These limitations reflect the challenges of assessing patients presenting acutely with severe ventilatory failure but should be considered when interpreting the findings.

## Conclusions

This case demonstrates that acute hypercapnic respiratory failure may represent the first manifestation of previously unrecognized ALS. When severe hypercapnia occurs without clear intrinsic pulmonary pathology, clinicians must recognize the diagnostic mismatch between an unremarkable chest examination and profound arterial blood gas abnormalities. Consequently, a targeted history and focused neurological assessment are crucial for identifying subtle upper and lower motor neuron signs during acute presentations. Early recognition in emergency settings is vital to prevent diagnostic delays, optimize immediate critical care, and facilitate timely specialist referral.

Evidence from previous studies indicates that appropriately selected patients with ALS may derive survival and quality-of-life benefits from noninvasive ventilation. Early diagnosis also facilitates timely multidisciplinary care, including symptom management, nutritional support, respiratory follow-up, and advance care planning. Increasing awareness of this uncommon presentation may facilitate earlier diagnosis, timely ventilatory support, and appropriate specialist referral.
